# E-cigarette Aerosol Condensate Enhances Metabolism of Benzo(a)pyrene to Genotoxic Products, and Induces CYP1A1 and CYP1B1, Likely by Activation of the Aryl Hydrocarbon Receptor

**DOI:** 10.3390/ijerph16142468

**Published:** 2019-07-11

**Authors:** Yuan-Wan Sun, Wieslawa Kosinska, Joseph B. Guttenplan

**Affiliations:** 1 Department of Biochemistry and Molecular Biology, Pennsylvania State University, Hershey, PA 17033, USA; 2 Cancer Institute, Pennsylvania State University, Hershey, PA 17033, USA; 3 Department of Basic Science, New York University College of Dentistry, New York, NY 10010, USA; 4 Department of Environmental Medicine, New York University School of Medicine, New York, NY 10019, USA

**Keywords:** E-cigarette, aerosol, benzo(a)pyrene, CYP1A1, CYP1B1, aryl hydrocarbon receptor

## Abstract

E-cigarette aerosol contains lower levels of most known carcinogens than tobacco smoke, but many users of e-cigarettes are also smokers, and these individuals may be vulnerable to possible promoting and/or cocarcinogenic effects of e-cigarettes. We investigated the possibility that a condensate of e-cigarette aerosol (EAC) enhances the metabolism of the tobacco carcinogen, benzo(a)pyrene (BaP), to genotoxic products in a human oral keratinocyte cell line. Cells were pretreated with EAC from two popular e-cigs and then with BaP. Metabolism to its ultimate carcinogenic metabolite, anti-7,8-dihydroxy-9,10-epoxy-7,8,9,10-tetrahydro B[a]P (BPDE), was assayed by measuring isomers of its spontaneous hydrolysis products, BaP tetrols. The pretreatment of cells with EAC enhanced the rate of BaP tetrol formation several fold. Pretreatment with the e-liquid resulted in a smaller enhancement. The treatment of cells with EAC induced CYP1A1/1B1 mRNA and protein. The enhancement of BaP tetrol formation was inhibited by the aryl hydrocarbon receptor (AhR) inhibitor, α-napthoflavone, indicating EAC likely induces CYP1A1/1B1 and enhances BaP metabolism by activating the AhR. To our knowledge, this is first report demonstrating that e-cigarettes can potentiate the genotoxic effects of a tobacco smoke carcinogen.

## 1. Introduction

E-cigarette (e-cig) usage has become widely prevalent in the U.S. and other countries. There is considerable controversy as to the potential cancer risks and benefits posed by e-cigs [1,2]. Although studies on e-cig liquids, aerosols and condensates have indicated that they contain much lower levels of known carcinogens than tobacco or tobacco smoke [3], there is little reported research on the effects of e-cig usage on cancer risk in current tobacco users (dual users). A large 2016 study reported a prevalence of 4.5% of e-cig users above 18 years of age in the U.S., corresponding to approximately 10.8 million adult users and the prevalence was approximately twice as high in the 18–24 year old subpopulation, strongly suggesting that e-cig use will increase in the near future [4,5]. Importantly, a high percentage of younger users are dual users [4,5]. In such users, oral cavity tissue will be exposed to the genotoxic effects of tobacco smoke and may be susceptible to promoting or cocarcinogenic effects of other agents, such as e-cigs. Tobacco smoking is a major contributor to oral cancer and smokers have a much higher risk for developing oral cancer than nonsmokers [6]. E-cig aerosols have lower levels of most known tobacco genotoxic carcinogens than tobacco smoke, with the exception of formaldehyde and perhaps other small aldehydes [3,7,8]. However, it seems important to determine whether e-cig usage can impact the potential carcinogenicity of tobacco smoke by mechanisms other than genotoxicity. We therefore examined the effect of e-cig aerosol condensates (EACs) on the metabolism by human oral cells of a major known tobacco carcinogen, benzo(a)pyrene (BaP).

Carcinogenic polycyclic aromatic hydrocarbons such as BaP require metabolic activation in order to exert genotoxic effects [9,10,11]. BaP is converted to its ultimate genotoxin, anti-7,8-dihydroxy-9,10-epoxy-7,8,9,10-tetrahydro B[a]P (BPDE), in several steps initiated by cytochrome P-450 1A1 and/or 1B1 [9,10,11]. We observed that EAC significantly enhanced the rate of metabolism of BaP to genotoxic products and induced the expression of cytochrome P-450s 1A1 and 1B1, likely via the activation of the aryl hydrocarbon receptor (AhR). To our knowledge, this is the first report that e-cigs enhance the rate of metabolism of a tobacco carcinogen. 

## 2. Materials and Methods

Keratinocyte Growth Medium (KGM) was obtained from Lonza Bioscience (Walkersville, MD, USA). BaP, α-napthoflavone and nicotine liquid were from Sigma-Aldridge (St. Louis, MO, USA). BPDE was obtained from the Chemical Carcinogen Reference Standard Repository, Midwest Research Institute (Lexena, KS, USA). 

### 2.1. Cell Line and Culture Conditions 

MSK Leuk1 cells were established from a premalignant leukoplakic lesion adjacent to a squamous cell carcinoma of the tongue [12]. The cells were obtained from Peter Sacks, who is an emeritus faculty member in the same department as J.B.G. The cells were authenticated by Genetica DNA Laboratories (Burlington, NC, USA) using short tandem repeat DNA profiling. Sequencing studies indicated that a GC > AT transition in exon 8 in one allele of p53, resulting in a glu to lys mutation in codon 286, was present in the MSK Leuk1 cells [13]. This cell line was routinely maintained in KGM grown to 70% confluence, and trypsinized with 0.125% trypsin-2 mmol/L EDTA (Sigma-Aldrich, St. Louis, MO, USA) solution before passage. 

### 2.2. E-Cig Aerosol Condensate Preparation 

**Figure 1:**
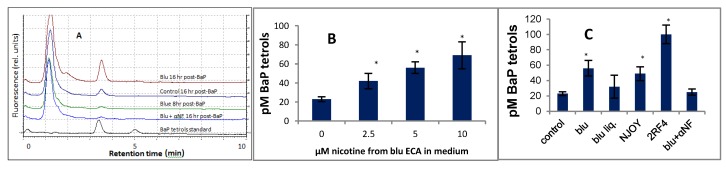
Effects of aerosol condensates (EACs) from blu and NJOY e-cigarettes, blu e-liquid, tobacco smoke extract (TSE), and α-napthoflavone on the metabolism of BaP to BaP-tetrols by MSK Leuk 1 cells. (**A**) Representative chromatograms of BaP tetrols. Cells were incubated for 18 h with or without additive and then for up to 16 h with 0.5 µM BaP; 50 µL of the filtered culture medium was applied to HPLC using conditions described in the Results and Discussion. The combined mass of the two standard BaP tetrols was 5 fmol. Traces (bottom to top), BaP tetrol I and II; tetrols generated from cells treated with BaP and: blu + α-NF (16 hr post BaP); blu (8 hr post-BaP); no additive (16 hr); blu (16 hr, post-BaP). (**B**) Dependence of the metabolism of BaP to BaP tetrols on the concentration of blu EAC. Concentrations of EAC are expressed as the concentration of nicotine they yield when added to the culture medium. The ordinate represents the concentration of BaP-tetrols generated in the culture medium. (**C**) Effects of EACs of blu, and NJOY, and TSE on the metabolism of BaP to BaP-tetrols. The concentration of nicotine in the medium was 5 uM for all of the EACs and e-liquid, and 2.5 uM for the TSE. The α-napthoflavone concentration was 10 uM. * *p* < 0.05 relative to the unpretreated control, using a two-tailed t-test.

**Figure 2:**
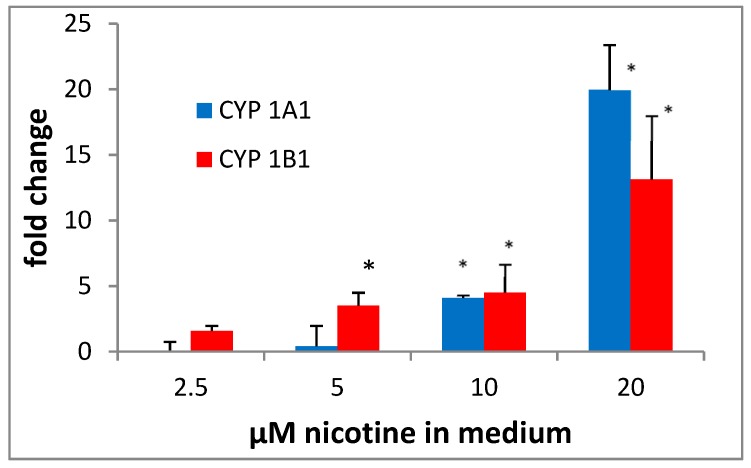
Effect of EAC from blu on CYP1A1 and 1B1 mRNA levels in MSK Leuk 1 cells. Cells were treated for 18 hr with the EAC such that every µL of blu extract yielded 2.5 µM nicotine/mL medium. RNA was then isolated, qPCR was performed, and ΔCt values were normalized to LDH. * *p* < 0.05 relative to the unpretreated control, using a two-tailed t-test.

**Figure 3:**
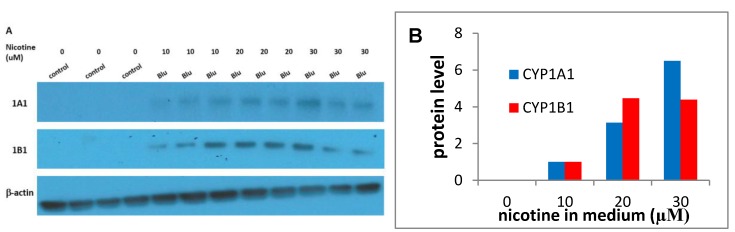
Effect of EAC from blu on CYP1A1 and 1B1 protein levels in MSK Leuk 1 cells. Cells were treated for 24 hr with the blu EAC and CYP1A1 and CYP1B1 protein levels were determined by Western blotting. (**A**) Blotting images; (**B**) Quantitation of images. The levels of CYP1A1 and CYP1B1 were below detection, so the levels of CYP1A1 and CYP 1B1 were normalized to results obtained at 10 uM nicotine, although the enhancement relative to the vehicle control is unknown.

A popular disposable e-cig (blu classic tobacco, 2.4% nicotine) was “smoked” using a programmable single port Aerosol Single Port Electronic Cigarette Generator pump (eAerosols, Central Valley, NY 10917, USA) to generate an IOS standard puff of 35 mL per minute using a 4 s duration at 30 s intervals. One experiment was also carried out with a disposable NJOY King (bold tobacco flavor, 4.5% nicotine), which was smoked the same way. The e-cig aerosol was connected via a 12-inch length of tygon tubing to a U-tube which was immersed in liquid nitrogen. The condensate was dissolved in 25% DMSO/water to a concentration of one mg/mL (6.17 mM) nicotine. This was further diluted with phosphate buffered saline (PBS) (Fisher Scientific, Pittsburg, PA, USA) and added to the culture medium to yield the concentrations of nicotine indicated in Figure 1, Figure 2 and Figure 3.

### 2.3. E-Cigarette Liquid 

We also tested the liquid in blu e-cigs (blu classic tobacco E-liquid, 2.4% nicotine) for its ability to enhance the rate of metabolism of BaP to BaP tetrols. For this experiment, we diluted the e-liquid in PBS, so it contained the same percentage of nicotine as the blu aerosol condensate.

### 2.4. Preparation of Tobacco Smoke Extract (TSE)

The preparation of the tobacco smoke extract was previously described [14]. Briefly the cigarette smoke was generated with an automated cigarette smoking machine (CH Technologies, Ewing, City, NJ, USA). An automatically regulated piston pump produced a two second puff of 35 mL volume (a standard used in U.S. smoke exposure studies). The smoke from one pack of 2RF4 Kentucky reference cigarettes was impinged onto a Cambridge filter (Fisher Scientific, Pittsburg, PA, USA) and particulates were extracted from the filters in acetone and diluted in PBS as necessary. The filters were weighed before and after particulates were extracted. 

### 2.5. Metabolism of BaP by MSK Cells

For the assays for the metabolism of BaP to BaP tetrols, cells were seeded into CytoOne 96-well cell culture dishes (USA Scientific, Orlando, FL, USA) at a density of 20,000 cells/well in 100 µL of medium. On the following day, cells were treated overnight with (1) aerosol condensates of blu and NJOY (New York, NY, USA), (2) BLU e-liquid (Fontem Ventures, B.V., Amsterdam, The Netherlands) or (3) TSE at concentrations indicated in Figure 1B,C and then for several time periods up to 16 hr with 0.5 µM BaP. Each measurement was performed in triplicate. 

### 2.6. Gene Expression

For mRNA gene expression and immunoblotting experiments, cells were treated in 6-well CytoOne cell culture dishes and grown to approximately 80% confluence. For mRNA isolation, cells were treated as described in Figure 2 and harvested 16 hr later. For immunoblotting, cells were harvested 20 h after treatment. qPCR Primer pairs were obtained from Sigma (KiCqStart™ Primer pair H_CYP1A1_2 and H_CYP1B1_1) (St. Louis, MO, USA).

### 2.7. Analysis of BaP Tetrols

For BP tetrol analyses, aliquots of the culture medium were eluted from a Keystone Hypersil C18 (Fisher Scientific, Pittsburg, PA, USA) 3 µ 3 × 50 mm column in a mobile phase of 30% acetonitrile/water at a flow rate of 0.4 mL/min. The eluate was analyzed using the above HPLC column with a fluorescence detector set at 344-nm excitation and 400-nm emission. A Shimadzu (Kyoto, Japan) high-performance liquid chromatography system consisting of an LC-20AD solvent delivery system, a SIL-10Ai autoinjector, and an RF-10AxL fluorescence detector was used for analysis. Quantitation of the tetrols was achieved by comparison with standards of the B[a]P tetrol isomers. These were generated by incubating anti-BPDE in water at room temperature for one hr. The tetrol designated BPDE tetrol I-1 (1) [14,15] was the major one produced in the cultured cells. Only trace amounts of the minor adduct, BPDE tetrol I-2, were detected. 

### 2.8. Analysis of Nicotine

Nicotine concentration in the EACs was by determined by HPLC using a Thermo BetaBasic-18 (Fisher Scientific, Pittsburg, PA, USA), 50 × 4.6 mm 3 µ particle size HPLC column, with an isocratic 0.4mL/min flow rate and a mobile phase of 5 mM sodium phosphate in 30% acetonitrile containing 6% SDS at a pH of 2.2. 

### 2.9. mRNA Expression

RNA was isolated using a Promega SV total RNA isolation kit (Fisher Scientific, Pittsburg, PA, USA). RNA was subjected to 1% agarose gel electrophoresis, showing two tight bands at 28 S and 18 S, with the 28 S band approximately twice the intensity of the 18 S band. RNA was converted to cDNA with a Verso RT² First Strand kit from ThermoFisher (Pittsburg, PA, USA). ΔCt values were obtained relative to LDH. Each measurement was performed in triplicate.

### 2.10. Western Blot Analysis

Equal amounts of protein (10 µg) were diluted with 2× sample buffer containing β-mercaptoethanol (Sigma-Aldrich) and bromophenol blue (Sigma-Aldrich) and heated at 100 °C for 5 min. Proteins were separated by SDS-PAGE with 10% acrylamide followed by transferring onto PVDF membranes. Membranes were then blocked with 5% nonfat dry milk (Bio-Rad, Hercules, CA, USA) and probed overnight in the same blocking solution at 4 °C containing antibodies against CYP1A1 (Abcam, cat#ab79819, Cambridge, MA, USA), CYP1B1 (Abcam, Cat#ab185954) or β-actin (Sigma-Aldrich, cat#A2228). Immunoreactive proteins were subsequently incubated with appropriate secondary antibodies conjugated with HRP and visualized using enhanced chemiluminescence reagents (Thermo Fisher scientific, Waltham, MA, USA). After film development, the densitometric value was determined using GS800 Calibrated Densitometer (Bio-Rad Laboratories, Hercules, CA, USA) and quantified with the Quantity One v4.5.0 1D analysis software (Bio-Rad Laboratories). The relative densities of 1A1and 1B1 to β-actin were calculated. 

## 3. Results

The effects of blu, and NJOY aerosol condensates, and blu e-liquid on the metabolism of BaP are discussed in this section. BaP is metabolized to a number of products, including isomers of the highly reactive BPDE, which can bind to guanines in DNA and lead to mutations [9,10,11]. BPDE can also spontaneously hydrolyze to isomers of 7,8,9,10-tetrahydroterahydroxy-7,8,9,10-BaP (BaP tetrols) [15]. In cells metabolizing BaP, these tetrols can be released into the medium. In a previous study, we have shown that there is an excellent linear correlation between relative DNA adduct levels and relative BP tetrol concentrations in the cell medium when DNA adducts and BaP tetrols are assayed in cells and medium taken at the same time [14]. This correlation holds at different concentrations of tobacco smoke extract [14]. Hence levels of BP-tetrols in the cell medium can serve as a surrogate for levels of BPDE DNA adducts. 

MSK cells were pretreated with EAC or TSE for 16 hr and BaP was then added, and the culture medium was analyzed for BP-tetrols at several time periods (Figure 1A). The concentrations of EAC or TSE in the medium resulted in nicotine levels approaching those of total nicotine + nicotine metabolites in the saliva of “vapers” or smokers [16,17].

Under the assay conditions employed, little product was detected at 8 hr, and 16 hr was chosen for the analysis time. At 16 hr, all treatments resulted in the enhanced production of BP-tetrols, relative to untreated cells, with the order of effectiveness being TSE > blu > NJOY > blu e-liquid (Figure 1A–C) under the treatment conditions described. The e-liquid from blu gold leaf liquid at a similar concentration of nicotine as in the aerosol condensates also enhanced tetrol formation, but under the conditions described, the enhancement did not reach statistical significance. 

### 3.1. Effect of α-Napthoflavone on the Metabolism of BaP

Activation of the AhR stimulates the transcription of CYP1A1 and CYP1B1, which encode proteins that convert polycyclic aromatic hydrocarbons to genotoxic metabolites [18]. The inhibition of this activation should then reduce the rate of formation of these metabolites. Alpha-napthoflavone, (αNF) a known antagonist of AhR, was then investigated as to whether it could inhibit the EAC-enhanced conversion of BaP to BP-tetrols in MSK cells (Figure 1A,C). Indeed, αNF was an effective inhibitor of the formation of BP-tetrols, (Figure 1A,C) suggesting that some component of EAC is capable of activating the AhR. 

### 3.2. Cyp1a1 and 1B1 

We thought it likely that the enhanced metabolism of BaP to tetrols was a result of the induction of CYP1A1 and/or 1B1 by EAC and TSE. Previous studies have shown that tobacco smoke preparations are effective inducers of CYP1A1 and 1B1 in cultured cells [19,20,21]. We first monitored the effect of a BLU on mRNA expression using qPCR (Figure 2). 

Consistent with the metabolism results, the EAC treatment resulted in a concentration-dependent increase in CYP1A1 and 1B1 mRNA. To confirm that expression was also enhanced at the protein level, we measured the effects of blu EAC by immunoblotting and observed an increase in the CYP1A1 and 1B1 protein levels (Figure 3A,B). 

## 4. Discussion

This study is the first to report that e-cig exposure stimulates the metabolism of a tobacco procarcinogen to its genotoxic metabolite in a precancerous cell line. The oral cavity of long-term smokers likely contains precancerous cells, due to continued exposure to genotoxins. This study also extends a previous study on the effects of e-cig exposure on the transcriptome of human bronchial cells that found enhancing effects on CYP1A1 [22]; and results reported in another study show that lung microsomes from e-cig-treated mice exhibited enhanced 7-methoxyresorufin O-demethylase activity. This activity reflects CYP1A1/2 [23]. It is significant that enhancement in the conversion of BaP to BaP-tetrols by EAC was observed, as the induction of CYP1A1/1B1 is a necessary but not sufficient condition for the activation of BaP to genotoxic products, as other processes (e.g., induction of phase II enzymes, competitive inhibition by inducers) can influence the ultimate effect of a CYP1A1/1B1 inducer on metabolism of procarcinogens to genotoxic products [24]. We observed that EAC in the culture medium at levels near those observed in the saliva of vapers induced at least the several-fold induction of CYP1A1 and 1B1at the RNA and protein levels. The actual induction of protein is uncertain because the levels of CYP1A1 and 1/1B1 were below the limit of detection under our assay conditions, but based on the differences in expression in going from 10 to 20 and 20 to 30 uM nicotine in medium, it seems likely that at 10uM nicotine, there is a 2–3-fold increase in expression relative to controls.

E-cigs are often touted as a safer alternative to tobacco smoking. While it is true that, other than formaldehyde, e-cig vapor contains lower levels of known carcinogens than tobacco smoke aldehydes [3,7,8], the results presented here suggest EAC could enhance the carcinogenic risk resulting from residual tobacco smoke particulates in the oral cavity and possibly other aerodigestive tissue by increasing the rate of conversion of PAHs to genotoxic products. Although tobacco smoke itself is a potent inducer of AhR, in dual users, after active tobacco smoking has ceased and the bulk of the tobacco particulates have been cleared, the levels of CYP1A1/1B1 will also decline; and under this condition, the inducing effects of EAC may become relatively more important in converting PAHs in residual particulates to genotoxic products. Alternatively, dual users who regularly use e-cigs and use tobacco on an intermittent basis will likely have the enhanced conversion of PAHs to genotoxic products on commencing tobacco smoking, before the induction of CYP1A1/1B1 by tobacco smoke. 

At the moment, the identity or nature of the AhR inducer in EAC is unknown. Many AhR agonists are polycyclic, hydrophobic and planer or near planer, but others, generally containing at least two aromatics, are known [25,26]. The major components of most e-cig liquids are some combination of propylene glycol, glycerin, and nicotine, but none of these major components are typical of AhR ligands. Generally, a small concentration of flavorant is also present; in the tobacco flavored e-cigs, this is usually vanillin, diacetyl or pentanedione [27], but these do not fit the above description. However, e-cigarettes are stored at room temperature and the aerosol is heated, and additional compounds may thus be generated. New glycol-derived products generated from aldehyde flavorants and glycols in e-cigs have been reported [28]. It seems likely that ketone flavorants could also form adducts with glycols, other favorants or themselves. In addition, e-cigs generate free radicals [29], which are highly reactive species. 

We observed EAC-enhanced BaP metabolism with two different brands of e-cigs, suggesting our observations are relevant to other types of e-cigarettes. With respect to the AhR, persistent activation of the AhR is thought to be a risk factor for carcinogenesis [25]. It appears that there is some CYP-450-inducing ability in e-liquid, but less so than in the aerosol condensate. However, we do not know how well the composition of the condensate reflects the e-liquid, as the efficiency of the trapping all of the components may not be equal and heating the aerosol may generate new compounds. 

## 5. Conclusions

In conclusion, EAC and possibly e-cig liquid, when added to cultures of human oral keratinocytes at levels approximating those in the saliva of e-cig users, are capable of enhancing the rate of the metabolism of BaP to genotoxic metabolites. It is likely that the enhanced metabolism of BaP and induction of CYP 1A1/1B1 results from activation of the AhR.
